# Borneol Attenuates Ultrasound-Targeted Microbubble Destruction-Induced Blood–Brain Barrier Opening in Focal Cerebral Ischemia

**DOI:** 10.3389/fneur.2017.00704

**Published:** 2017-12-22

**Authors:** Xiao-guang Zhang, Ye Song, Chang Shan, Xi-fan Wu, Yan-hua Tong, Xin-chun Jin, Wen-lan Liu, Guo-qing Zheng, Jie Liu

**Affiliations:** ^1^Department of Neurology, The Second Affiliated Hospital and Yuying Children’s Hospital of Wenzhou Medical University, Wenzhou, China; ^2^Translational Center for Stem Cell Research, Tongji Hospital, Stem Cell Research Center, Tongji University School of Medicine, Shanghai, China; ^3^Department of Ultrasound, Shanghai Tongji Hospital of Tongji University, Shanghai, China; ^4^Department of Endocrine and Metabolic Diseases, Shanghai Clinical Center for Endocrine and Metabolic Diseases, Rui-jin Hospital, Shanghai Jiao-tong University School of Medicine, Shanghai Institute of Endocrine and Metabolic Diseases, Shanghai, China; ^5^Jiangsu Key Laboratory of Translational Research and Therapy for Neuro-Psycho-Diseases and Institute of Neuroscience, The Second Affiliated Hospital of Soochow University, Soochow University, Suzhou, China; ^6^The Central Laboratory, Shenzhen Second People’s Hospital, The First Affiliated Hospital of Shenzhen University, Shenzhen, China

**Keywords:** borneol, unfocused, ultrasound-targeted microbubble destruction, ischemic stroke, blood–brain barrier

## Abstract

Ultrasound-targeted microbubble destruction (UTMD) and the herb medicine borneol can both facilitate the delivery of therapeutic agents to diseased brain regions and serve as promising adjuvant neuroprotective therapies. Our preliminary experiments showed that UTMD could exacerbate ischemic blood–brain barrier (BBB) opening, while borneol can protect the BBB. In this study, we tested the hypothesis that the combination of UTMD and borneol could attenuate UTMD-induced injury to the BBB under ischemic stroke conditions. Male albino mice were subjected to 60-min middle cerebral artery occlusion (MCAO) with reperfusion. Borneol and UTMD was given to mice 3 days before and 24 h after MCAO induction. BBB permeability, brain water contents, ultrastructural changes of the BBB and histopathological alterations were evaluated. Our data demonstrated that UTMD aggravated the leakage of Evans blue dye, ultrastructural alterations of cerebral microvasculature, brain edema, and even induced cerebral hemorrhage in ischemic stroke mice. Pretreatment with borneol significantly attenuated the above detrimental effects of UTMD on the BBB. This study indicates that under ischemic stroke conditions, the BBB becomes vulnerable to UTMD intervention, and the combination of borneol can help to maintain the integrity of the BBB.

## Introduction

Stroke is the second most common cause of death and the major cause of acquired disability worldwide (1). After several decades of research on neuroprotectants against ischemic stroke, few successful results have been obtained and no single neuroprotectant agent has been approved so far (2). The blood–brain barrier (BBB), a highly selective permeability barrier, separates the circulating blood from the central nervous system (CNS) and plays a pivotal role in maintaining brain homeostasis. BBB opening is frequently seen during cerebral ischemia and reperfusion (3), which could theoretically allow neuroprotectants to get into the affected brain tissue. However, targeted delivery of neuroprotectants may still be hindered, even by an ischemia-induced “open” BBB (4). Therefore, novel therapeutic strategies that can induce safe and more BBB opening are urgently needed for neuroprotective therapy in ischemic stroke.

Mounting evidence from animal studies has demonstrated that ultrasound-targeted microbubble destruction (UTMD) can noninvasively and selectively facilitate the delivery of therapeutic agents to specific brain area through transiently opening the BBB and is increasingly appreciated as a promising adjunct therapy for ischemic stroke (5, 6). Fatar et al. have demonstrated that UTMD significantly reduces the infarct volume and glutamate level in the brain of ischemic rats, supporting its neuroprotective role in acute ischemic stroke (7). Wang et al. reported that transcranial UTMD-mediated vascular endothelial growth factor plasmid delivery significantly reduced apoptosis and infarct size and improved neurologic function in ischemic stroke mice (5). While researchers are obsessed with exploring how UTMD effectively assists the neuroprotective agents in the treatment of CNS diseases, it has also been reported that UTMD’s mechanical and cavitation effects can cause normal brain tissue bleeding (8, 9). Our preliminary experiments also showed that UTMD at a setting that was safe and relatively modest for BBB opening in healthy mice could exacerbate BBB opening and result in erythrocytes extravasations in ischemic stroke mice. These findings raise an important safety concern for the clinical application of UTMD as an adjuvant post-stroke therapy.

Herb medicine borneol is a simple bicyclic monoterpene extracted from several species of Artemisia and Dipterocarpaceae. Acting as a good penetration enhancer, borneol remains to be one of the most common Chinese herbal medicine prescribed for treating cerebrovascular disease (10). Animal stroke studies have demonstrated that borneol treatment could effectively alleviate the alterations in BBB integrity including tight junctions (TJs) opening, basal lamina degradation, permeability increase, and brain edema following cerebral ischemia and reperfusion (11, 12). In addition, borneol has been reported to be neuroprotective *via* anti-apoptosis, scavenging intracellular reactive oxygen species, and maintaining normal mitochondrial membrane potential (13, 14). These multifaceted actions of borneol have prompted us to test the hypothesis that the combination of UTMD and borneol may serve as a safer BBB opening strategy than UTMD alone under ischemic stroke conditions.

In this study, we tested the above hypothesis in a mice model of middle cerebral artery occlusion (MCAO) with reperfusion and validate the role of borneol and UTMD in BBB permeability, ultrastructure of microvessels, and brain water content under ischemic states, which might have great significance for clinical treatment of ischemic stroke.

## Materials and Methods

### Animals

Male adult Institute of Cancer Research albino mice weighting 25–30 g were obtained from Shanghai Laboratory Animal Research Centre and housed in a temperature-controlled room (22.0 ± 2.0°C) under a 12 h light:12 h dark cycle and relative humidity of 55 ± 10%. Food and water were available *ad libitum*. All procedures associated with the care of animals were approved by the Animal Ethics Committee of Shanghai Tongji Hospital and performed according to the National Institutes of Health’s Guide for the Care and Use of Laboratory Animals, which was published in 1996. All efforts were made to reduce the number of animals used.

Animals were randomly divided into four groups: I/R group (*n* = 16), I/R + borneol group (*n* = 18), I/R + UTMD group (*n* = 18), and I/R + borneol + UTMD group (*n* = 18). Borneol was dissolved in 5% Tween 80 and given to mice by oral gavage at 200 mg/kg 3 days before MCAO induction. UTMD was administered 24 h after MCAO induction.

### Focal Cerebral Ischemia and Reperfusion Model

Focal cerebral ischemia and reperfusion (I/R) was induced by transient MCAO as previously described (15). Briefly, the mice were anesthetized by inhalation of 5% isoflurane and maintained with 2% isoflurane in a mixture of 70% N_2_O and 30% O_2_. Under an operating microscope, right common carotid artery (CCA), internal, and external carotid arteries were exposed through a neck incision. The right CCA was ligatured and the circulation in the right external and internal carotid arteries was temporarily interrupted with a 6-0 silk suture. An arteriotomy was performed in the CCA proximal to the carotid bifurcation. A monofilament nylon suture (Sensas, 80 µm diameter), with its front end coated by “thermomelting” glue, (4 mm long, 190 µm diameter, Jet Melt, Radiospares, Beauvais, France) was introduced *via* lumen of right CCA advanced into the right internal carotid artery to occlude the origin of the right middle cerebral artery. The filament was withdrawn 60 min after occlusion to permit reperfusion.

### Application of Transcranial UTMD

**Figure 1:**
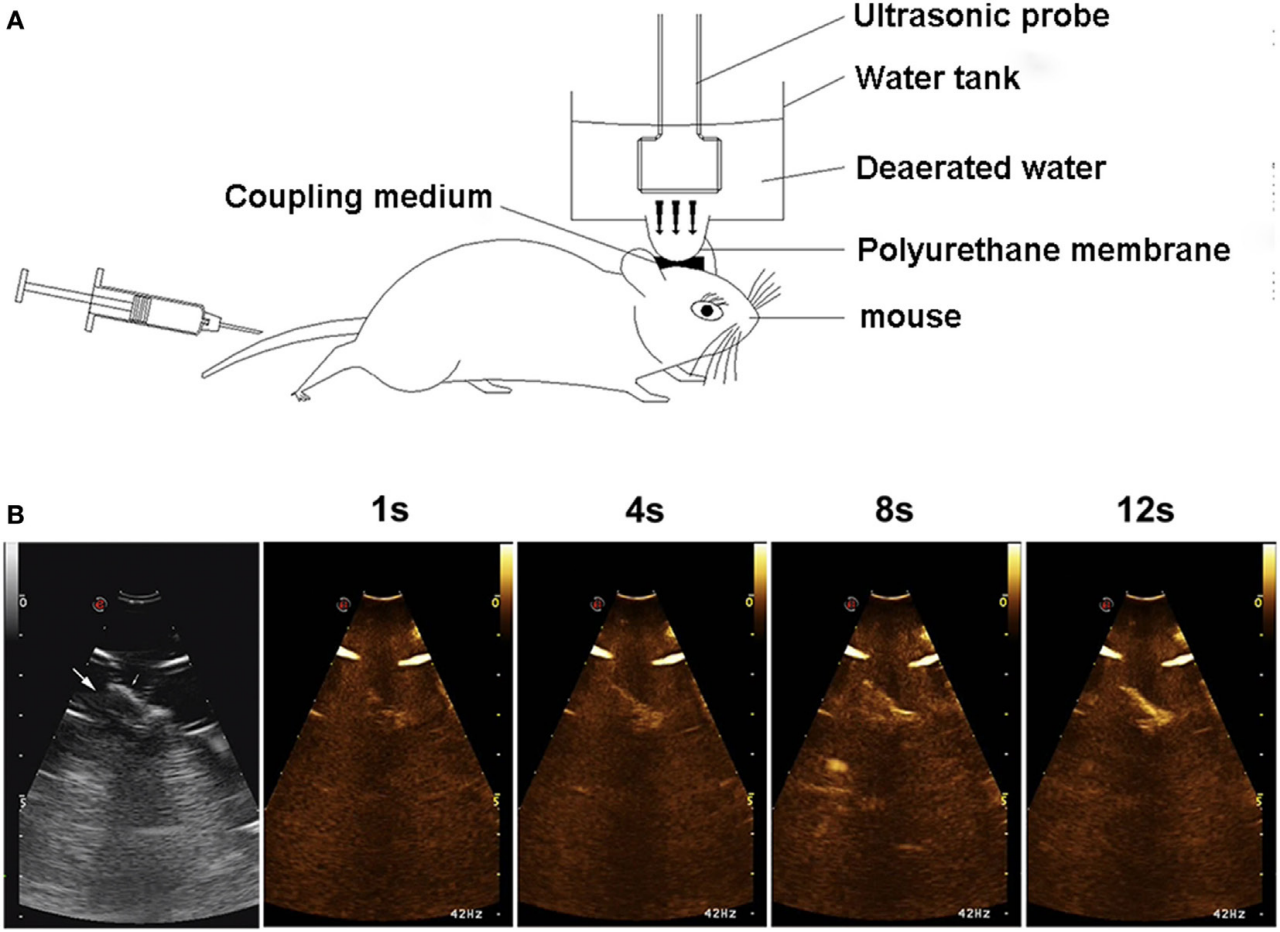
Experimental setup **(A)** and contrast-enhanced ultrasound (CEU) perfusion images of the right hemisphere **(B)**. Arrows show the brain tissue beneath skull. CEU signal increased with time and was mainly concentrated in the microvessels on brain cortex.

Mice were anesthetized by the intraperitoneal injection of 1% sodium pentobarbital (30 mg/kg). The hair over the skull was removed with an electric clipper and a depilatory cream. For sonification, the head of the mouse was immobilized with a stereotaxic equipment. The ultrasound probe (EC1123, Esaote Medical, Italy) was placed on the water container, between the scalp and the polyurethane membrane filling with ultrasound gel for the reduction of attenuation of ultrasound energy (Figure 1A). The transcranial UTMD was carried out with a diagnostic ultrasound apparatus (MyLabTwice, Esaote Medical, Italy), and simultaneously combined with an intravenous injection of SonoVue ultrasound contrast agent (Bracco) which was mixed with 5 ml normal saline before use. The mean diameter of microbubbles was 2.5 µm and their mean concentration was 5 × 10^8^/ml. The mice were treated with UTMD at the Flash mode (interval for 2 s), sonication frequency of 3.0 MHz, mechanical index (MI) 0.06/1.0, focus depth of 5 cm, volume of microbubble 0.1 ml, and duration 3 min. Throughout the process, the elliptical polyurethane membrane was held vertically in the right hemisphere in order to greater precision of the focus. Contrast-enhanced ultrasound perfusion was observed in the ultrasonic real-time angiography images (Figure 1B).

### Evaluation of BBB Permeability

Blood–brain barrier permeability was quantitatively evaluated by measuring Evans blue dye (EB) extravasation. EB (2% in saline, 4 ml/kg; Sigma-Aldrich) was intravenously injected immediately after the sonication. After 2 h, mice were anesthetized with isoflurane and were transcardially perfused with saline until the drainage from the right atrium became colorless. After decapitation, brains were coronally sectioned (15-μm thickness) using the cryostat (Leica CM1800, Heidelberg, Germany). Fluorescent images of the sections were acquired at cortex with fluorescence microscope (ECLIPSE 80i, Nikon, Japan) at a 20× magnification objective lens. Regional fluorescent intensity in the ipsilateral cortex was measured by using Image J (National Institute of Health, Bethesda, MD, USA).

Evans blue dye extravasation into the brain was measured as previously described (16). Samples were weighed and incubated in methanamide (Sigma-Aldrich). The supernatant was obtained by centrifugation and measured at 632 nm by Microplate reader (Multiskan MK3, Thermo, Finland). The amount of extravasated EB was expressed as micrograms per gram hemispheric tissue.

### Ultrastructure of Cerebral Microvessls

The mice were transcardially perfused with 4% glutaraldehyde in phosphate buffer saline (PBS) under deep anesthesia. For transmission electron microscopy (TEM), zones of the compromised BBB were cut into small pieces (<1 mm^3^) and rapidly fixed in 2.5% glutaraldehyde, then rinsed with 0.1 M PBS for three times, the tissue block was postfixed in 1% osmium tetroxide and dehydrated in ascending series of alcohol and embedded in araldite. Sections were processed with an ultramicrotome, stained with uranyl acetate and lead citrate, and examined in a TEM (JEM 1230, JEOL, Tokyo, Japan).

### Hematoxylin and Eosin Staining

The mice were killed by anesthetic overdose with 5% isoflurane and then perfused transcardially with saline followed by 4% paraformaldehyde. The brains were postfixed in the same fixative for 6–8 h and then dehydrated with 30% sucrose for 48 h. Coronal sections of 10 µm were processed with a cryostat and stained with hematoxylin and eosin.

### Examination of Brain Water Content

Cerebral water content was measured with the dry–wet weight method. Briefly, brains were rapidly removed and separated into the left and right cerebral hemispheres and immediately weighed (wet weight). Right brain specimens were then placed in an oven, dehydrated at 120°C for 48 h and weighed again (dry weight). The percentage of water content was calculated as follows: brain water content (%) = (wet weight − dry weight)/wet weight × 100%.

### Statistical Analysis

Measurement data were expressed as mean ± SD. Normality was determined using the Kolmogorov–Smirnov test. If normality was given and there were no significant differences in variance between groups (*F* test), multiple groups were compared using one-way analysis of variance and followed by LSD *post hoc* comparisons when appropriate. Otherwise, comparisons for non-normally distributed data were performed using nonparametric Kruskal–Wallis test followed by Mann–Whitney *U* test. All statistical analyses were performed with SPSS 20.0 software (IBM, Armonk, NY, USA) and GraphPad Prism 6.05 (GraphPad, La Jolla, CA, USA). *p* < 0.05 was considered statistically significant.

## Results

### Borneol Reduced UTMD-Induced EB Extravasation

**Figure 2:**
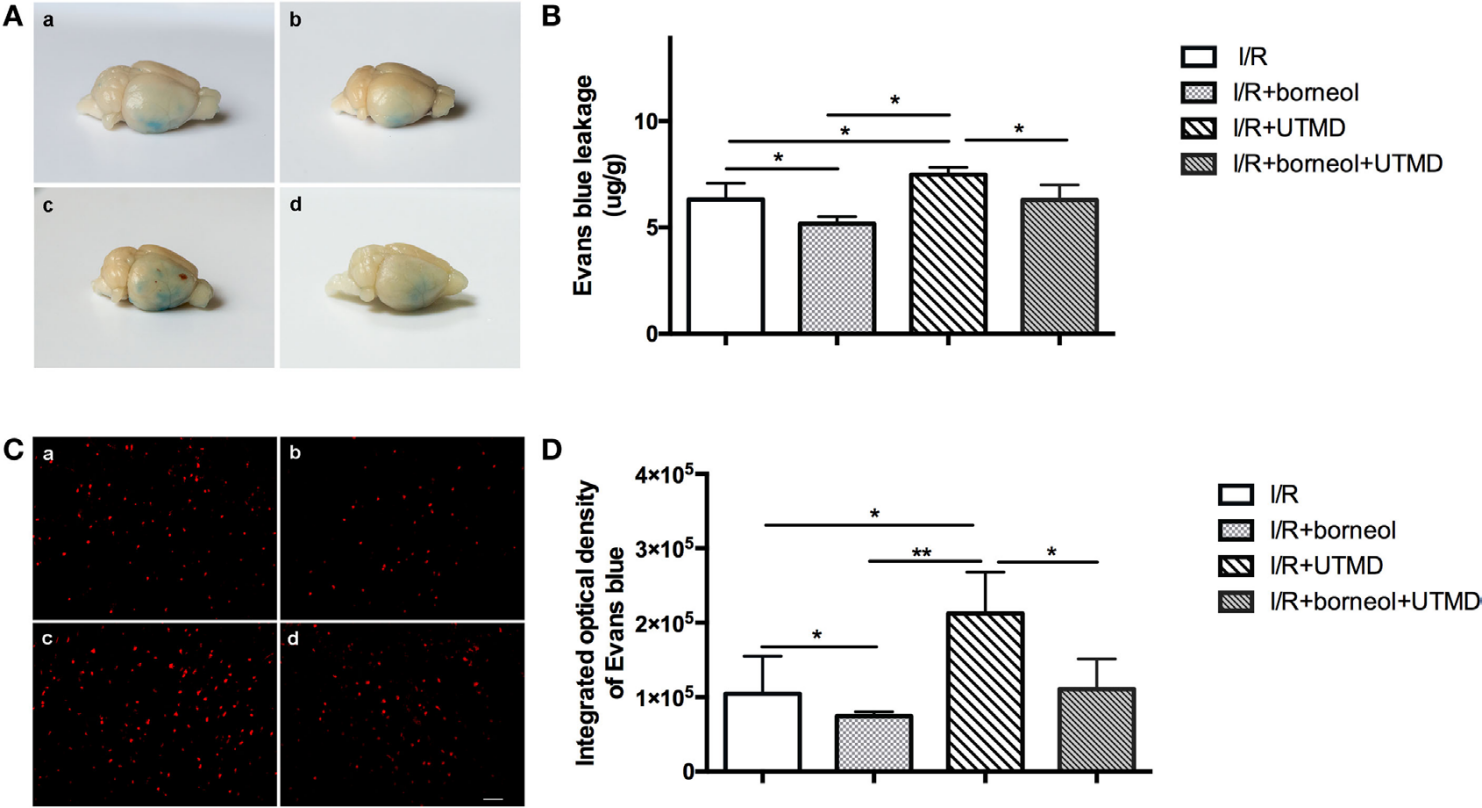
Borneol reduced ultrasound-targeted microbubble destruction (UTMD)-induced Evans blue dye (EB) extravasation. **(A)** Representative images of EB leakage in the brain surface among all groups. **(B)** The contents of EB leakage were measured in the right hemispheres among all groups, and expressed as micrograms per gram brain tissue, *n* = 5 per group. **(C)** Cortical fluorescence detection of EB in the right hemispheres among all groups, *n* = 3 per group. Scale bar = 50 µm. **(D)** Statistical analysis for fluorescence density of EB. (a) I/R group, (b) I/R + borneol group, (c) I/R + UTMD group, and (d) I/R + borneol + UTMD group. Data were represented as mean ± SD. *p < 0.05, **p < 0.01.

Blood–brain barrier integrity was evaluated by detecting EB extravasation at the ischemic (right) cerebral hemisphere of stroke mice. As shown in Figure 2, EB extravasation was significantly increased in I/R + UTMD group (7.48 ± 0.34) comparing to I/R group (6.32 ± 0.75) (*p* < 0.05). Borneol treatment attenuated EB extravasation for both I/R and I/R + UTMD groups, with the amount of EB extravasation reaching to 5.17 ± 0.34 and 6.29 ± 0.72, respectively (*p* < 0.05) (Figure 2). Similar results were obtained when EB extravasation was quantitated by measuring the fluorescence intensity of EB in ischemic cortical tissue, and the fluorescence intensities were 104,584 ± 50,558, 74,602 ± 6,019, 212,477 ± 55,587, and 110,803 ± 40,541 for I/R group, I/R + borneol group, I/R + UTMD group, and borneol + I/R + UTMD group, supporting a detrimental effect of UTMD while a protective effect of borneol on BBB opening under our experimental conditions (Figure 2).

### Borneol Attenuated the Alterations in Cerebromicrovascular Ultrastructure

**Figure 3:**
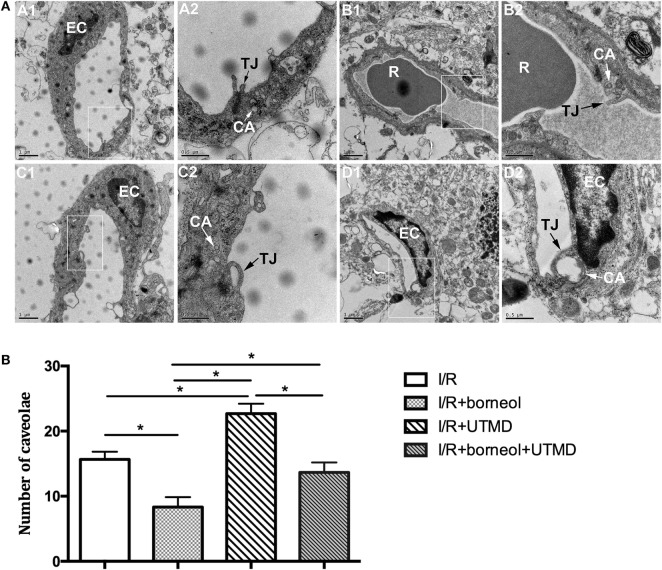
Borneol attenuated the alteration in ultrastructure of cerebral microvessels. **(A)** Representative transmission electron micrographs of capillaries in the cerebral cortex in different groups: (A1) I/R group; (B1) I/R + borneol group; (C1) I/R + UTMD group; and (D1) I/R + borneol + UTMD group. The micrograph in two is the high magnification of the area inside the box in 1, *n* = 3 per group. **(B)** Statistical analysis for number of caveolae. EC, endothelial cell; TJ, tight junction; R, red blood cell; CA, caveolae; UTMD, ultrasound-targeted microbubble destruction. Data were represented as mean ± SD. *p < 0.05.

Transmission electron microscopy was used to identify the ultrastructural alterations of cerebral microvasculature. As shown in Figure 3, ischemic cerebral microvessels of stroke mice showed perivascular edema, narrowed lumen, and disrupted TJs. Moreover, the basement membrane of affected capillaries was ruptured and discontinuous, and the endothelial cells (ECs) showed increased number of caveolae in the cytoplasm. UTMD intervention resulted in an augmented microvascular impairment, as reflected by more opening of TJs and more caveolae-like vesicles in the cytoplasm of ECs. Of note, the stroke mice receiving borneol treatment exhibited less severe alterations in the microvessels, further supporting a role borneol in BBB protection under ischemic stroke conditions.

### Borneol Reduced UTMD-Induced Cerebral Hemorrhage

**Figure 4:**
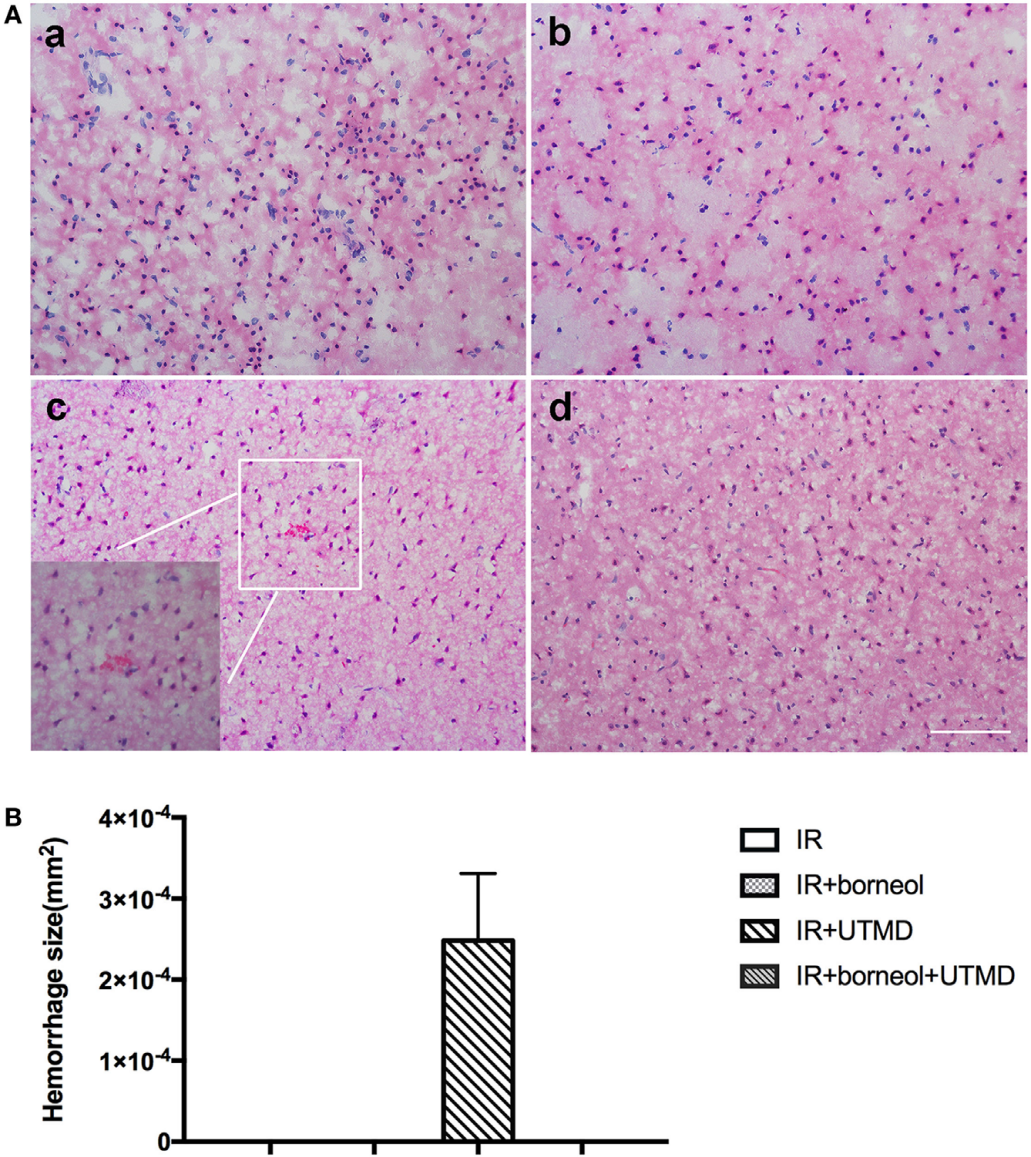
Borneol reduced ultrasound-targeted microbubble destruction (UTMD)-induced cerebral hemorrhage. **(A)** Representative photomicrographs of brain sections stained by hematoxylin and eosin: (a) I/R group; (b) I/R + borneol group; (c) I/R + UTMD group; and (d) I/R + borneol + UTMD group. **(B)** Statistical analysis for hemorrhage size. UTMD aggravated intracranial hemorrhage while borneol reduced its risk. *n* = 5 per group, scale bar = 50 µm. Data were represented as mean ± SD.

Hematoxylin and eosin staining was conducted to detect histological alterations in the ischemic cortex of experimental animals. As shown in Figure 4, conspicuous morphological changes were observed in all the four groups, including neuronal cell loss, pycnotic nuclei, and dark staining of the neurons. Besides remarkable cell damages, several erythrocytes were also found in the brain parenchyma of the ischemic hemisphere of I/R + UTMD mice, which was significantly reduced by borneol (Figure 4). These data indicate that the combination of borneol with UTMD could reduce the risk of cerebral hemorrhage under stroke conditions.

### Borneol Attenuated UTMD-Augmented Brain Edema

**Figure 5:**
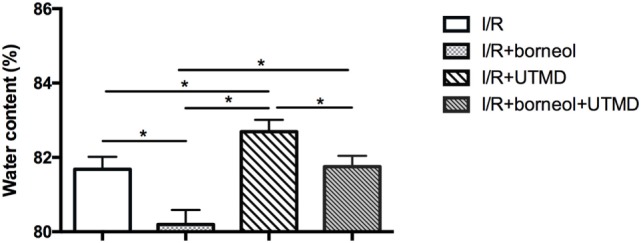
Borneol protected against the increase of water content caused by ultrasound-targeted microbubble destruction (UTMD). Each column represents quantification of brain water content. UTMD significantly increase brain water content while borneol protected against this increase, *n* = 5 per group, *p < 0.05.

We next evaluated brain edema by measuring brain water contents. As shown in Figure 5, UTMD intervention significantly increased the water contents in the ischemic hemisphere of the stroke mice (82.69 ± 0.32 for I/R + UTMD *vs* 81.68 ± 0.34 for I/R, *p* < 0.05), and borneol treatment effectively attenuated this increase (81.75 ± 0.29 for borneol + I/R + UTMD *vs* 82.69 ± 0.32 for I/R + UTMD, *p* < 0.05).

## Discussion

Our study demonstrated that UTMD intervention aggravated the leakage of EB, ultrastructural alterations of ischemic cerebral microvessels, brain edema and could even cause cerebral hemorrhage in ischemic stroke mice. When combined with herb medicine borneol, UTMD’s detrimental effect on the BBB was abolished, indicating that combining borneol with UTMD may help the BBB, in particular the ischemic BBB, to safely stand UTMD intervention.

Numerous studies have demonstrated that low frequency focused ultrasound with microbubbles could open the BBB and this technology was widely used for targeted delivery of therapeutic agents in recent decades (17, 18). But for the treatment of diseases with lesions widely distributed throughout the brain, a global therapeutics is needed. Thus, unfocused ultrasound, generating from a clinical diagnostic ultrasound instrument, was used in our study. The reason for choosing the transient I/R model, but rather than permanent occlusion, was because the former model was more close to the actual status of recanalization therapy for human ischemic stroke, where a large amount of stroke damage occurs during the phase of postischemic reperfusion (3). The reason for choosing the real-time flash ultrasonic imaging mode was because this model could not only allow us to clearly watch the process of contrast agent perfusion in the ultrasonic two-dimensional images, but could also keep the microbubbles for a longer time with a low MI (0.06); however, a burst of high MI (1.0) ultrasound could destroy the microbubbles during inertial cavitation (19). This flash technique has been previously applied for acute intravascular thrombi treatment and been demonstrated to be a safe operation (20). However, so far, few reports have addressed the safety issue of UTMD intervention in ischemic stroke. Our data show that UTMD treatment exacerbates ischemic BBB opening and even causes cerebral hemorrhage in the ischemic brain, highlighting the importance of seeking new strategies that can protect the BBB and reduce the risk of hemorrhage for UTMD intervention for ischemic stroke.

Our data that EB extravasation is evidently increased in the ischemic hemisphere indicate that cerebral ischemia and reperfusion result in serious BBB disruption. From the brain surface, EB is mainly confined to the bottom of temporal lobe in I/R group. Of note, UTMD treatment significantly expands the range of EB extravasation. In some of the stroke mice, there are visible bleeding spots on the brain surface too. These data indicate that UTMD can exacerbate BBB injury under ischemic stroke conditions, and it may even induce life-threatening brain edema or cerebral bleeding (21). When UTMD intervention is combined with borneol, the EB (blue-staining) areas are significantly smaller and very few bleeding spots are seen in the ischemic hemisphere, indicating that borneol can effectively attenuate UTMD-induced BBB opening. Considering the fact that borneol acts as a penetrating enhancer and can increase drug penetration into the brain, our data raise an important possibility that borneol, attributing to its BBB protective action, may serve as an effective adjunct therapy for facilitated drug delivery to the CNS by UTMD. In this study, we did not further explore the mechanism by which borneol protects the BBB.

Microbubbles expansion and contraction caused by ultrasound wave radiation generate a mechanical force impinged on the blood vessel wall, which can result in the opening of TJs (22). Here our data also show that UTMD applied during the phase of postischemic reperfusion induces remarkable endothelial damage at the ischemic cerebral microvessels. Similarly, borneol treatment effectively attenuates UTMD-induced ultrastructural change of the BBB. Interestingly, UTMD intervention increases the number of caveolae in the cytoplasm of capillary ECs. Caveolae has the properties of specific binding and is implicated in intracellular trafficking (23). Increased number of caveolae in the ECs after the application of UTMD is considered as the ultrastructural evidence for augmented transcellular transportation and is associated with high BBB permeability (24, 25). In addition, caveolin-1, the main structural component of caveolae, can combine with neuronal nitric-oxide synthase to inhibit excessive NO production in affected neuronal tissue, and thus plays an indirect role in brain protection (26). From this point of view, caveolae accumulation in endothelial cytosol of the BBB might act as a defensive machinery to help the BBB stand UTMD intervention. The effect of borneol on caveolae was likely to depend on the attenuated production of NO.

Our data that UTMD intervention augments I/R-induced BBB opening and may even increase the risk of cerebral hemorrhage is inconsistent with a previous study in which ultrasound combining microbubble did not cause extra damage to the brain in a rat model of ischemic stroke (7). The inconsistence may be due to lack of the histological observation in the previous study, or due to different animal species or experimental regime. In the previous study, UTMD was given during the phase of cerebral ischemia (3 h after ischemia onset). However, in our study, UTMD is applied at 24 h after cerebral onset based on the consideration of the fact that many conventional neuroprotectants are given at 24 h after stroke onset to reduce tissue damage caused by postischemic reperfusion (27, 28). This inconsistency suggests that UTMD may satisfy some therapeutic needs in stroke conditions where the BBB opening is relatively mild, while it may not be safe when the BBB is seriously damaged due to prolonged ischemia and reperfusion.

Blood–brain barrier opening and subsequent leaking out of serum proteins from the blood into the brain lead to vasogenic edema, which increases the brain water content (29). Our data show that UTMD markedly increases brain water contents in ischemic brain and borneol prevents this increase, further supporting that borneol could act as a BBB protecting agent to enhance the safety of UTMD intervention under ischemic stroke conditions.

There are several limitations in our study. First, this study is mainly focused on BBB opening, while how UTMD and borneol affect ischemic neuronal tissue damage and neurological function is not studied. Second, we only studied one stroke condition, i.e., 60-min ischemia with 24-h reperfusion, and whether and to what extent the combination of borneol and UTMD can protect the BBB in an earlier stage of ischemia and reperfusion or during permanent cerebral ischemia remains to be studied. Third, although we identified the definite protective effect of borneol on cerebral infarction, the direct impact of borneol on UTMD-associated BBB opening needed to be further verified. Lastly, we did not design experiments to test the effects of the combination of these two treatments on drug delivery to brain tissue under stroke conditions.

In summary, this study demonstrates that under ischemic stroke conditions, the BBB becomes vulnerable to UTMD intervention, and the combination of borneol can help to maintain the integrity of the BBB.

## Ethics Statement

This study was carried out in accordance with the recommendations of “National Institutes of Health’s Guide for the Care and Use of Laboratory Animals.” The protocol was approved by the “Animal Ethics Committee of Shanghai Tongji Hospital.”

## Author Contributions

G-qZ and JL conceived and designed the experiment. X-gZ, YS, CS, X-fW, Y-hT, and X-cJ performed the experiments. X-gZ analyzed and interpreted the data. X-gZ, CS, and JL wrote the article. W-lL revised the article critically for important intellectual content. All authors read and approved the final manuscript.

## Conflict of Interest Statement

The authors declare that the research was conducted in the absence of any commercial or financial relationships that could be construed as a potential conflict of interest.
